# Impact of perinatal HIV exposure and infection on salivary properties among Nigerian children

**DOI:** 10.1186/s12903-024-04159-z

**Published:** 2024-04-16

**Authors:** Nonso E. Onyia, Esosa Osagie, Paul Akhigbe, Nosakhare L. Idemudia, Ozo Obuekwe, Augustine Omoigberale, Vincent Richards, Modupe O. Coker

**Affiliations:** 1 Department of Oral Pathology Oral Medicine Oral Diagnosis, University of Calabar, Calabar, Cross River State Nigeria; 2 Research Department, Institute of Human Virology, Abuja, Nigeria; 3 Medical Microbiology Division, Medical Laboratory Services, University of Benin Teaching Hospital, Benin City, Nigeria; 4 Department of Oral and Maxillofacial Surgery, University of Benin, Benin City, Nigeria; 5 Institute of Child Health, University of Benin, Benin City, Nigeria; 6 Department of Biological Sciences, Clemson University, Clemson, SC USA; 7 Department of Oral Biology, Rutgers University, Newark, NJ USA

**Keywords:** Salivary pH, Salivary flow rate, Dental Caries, Perinatal, HIV exposure

## Abstract

**Background:**

There is growing evidence that perinatal HIV infection and exposure affect **s**alivary pH and flow rate in children in most parts of the world, but not against the background of caries and the African demographic. This study aimed to evaluate the impact of HIV infection as well as exposure on salivary properties and their influence upon the dental caries experience among school-aged children in Nigeria.

**Method:**

This cross-sectional study assessed the salivary flow rates and salivary pH of HIV infected and exposed school-aged (4–11) children receiving care at a Nigerian tertiary hospital. A total of 266 consenting participants which comprised of three groups as follows: (1) HIV Infected (HI) (*n* = 87), (2) HIV Exposed and Uninfected (HEU) (*n* = 82) and (3) HIV Unexposed and Uninfected (HUU) (*n* = 97) were recruited for the study. Questionnaires completed by parents/guardians were used for data collection. Three calibrated dentists performed oral examinations for dental caries. International Caries Detection and Assessment Scores (ICDAS) was used and presented as dmft/DMFT. Salivary pH was measured using MColourpHast™ pH indicator strips, while salivary flow rate was determined by collecting unstimulated whole saliva using the suction method. Data analysis relied on comparative statistics to determine the correlation between HIV exposure and infection on salivary pH and flow rates.

**Result:**

Across the groups, (HI, HEU, and HUU) mean pH of the HI was significantly less than that of HEU and HUU. Similarly, there was a statistically significant difference in the SFR across the three groups (*p* = 0.004). Other variables such as gender, age and oral hygiene status expressed by the gingival inflammatory scores had no significant influence on the pH and SFR of study participants. There was a rather unexpected positive correlation of DMFT of HI and HEU groups with increasing salivary flow rate; though, the relationship was weak and not significant.

**Conclusion:**

Perinatal HIV exposure and infection significantly impact salivary pH and flow rate among school-aged children in Nigeria. The findings of this study imply that HIV infection influenced the salivary pH, while HIV maternal exposure (without infection) impacted salivary flow rates when compared to the controls.

## Introduction

Dental caries has been categorized among diseases with complex and multifactorial aetiology with no single causative mechanism [1]. Its aetiology involves host, diet, oral microbiome, and salivary properties over time, resulting in demineralization of the tooth enamel leading to cavitation [2, 3]. For over seven decades, the Decayed, Missing and Filled Teeth (DMFT) index has been used globally as the most significant index for assessing oral and dental health status. This index determines the number of decayed, treated, and missing teeth due to decay, and while it is regarded as the most important index used in epidemiological studies, it aids in developing monitoring and evaluating oral health policies and interventions [4, 5]. The burden of oral disease in children living with HIV has been vigorously researched in the past decades. It is well documented that they have a significantly higher prevalence of dental caries, salivary gland and periodontal diseases than their uninfected counterparts [6–9]. Even though the oral health status of the rising population of HIV-exposed but uninfected (HEU) children remains scanty, especially in a sub-Saharan nation like Nigeria, some studies have reported the use of DMFT to evaluate the caries experience of children and adolescents exposed to and infected with HIV [10–12, 14, 15]. Interestingly, previous reports have described vulnerabilities of this special group, the HEU children. Generally, they have poorer health outcomes, an overall perturbed growth and are more likely to become stunted and underweight [13, 14]. They also have higher morbidity from infectious diseases, [15, 16] lack of parental care and immune abnormalities [17]. Findings from our group [18] and others [19–21] suggest little or no difference in caries experience comparing HEU group to HUU group. However, they appear to have a lower burden of caries compared to CLWH [19]. Nevertheless, additional studies are required to elucidate the impact of perinatal HIV exposure (not infection) on dental caries in young children.

During early and late stage of HIV infection, both adults and children, manifest clinical reduction in salivary flow rates of the major salivary glands [22]. Even though salivary gland hypofunction and saliva properties alteration is seen in people with HIV infection, suggesting that HIV infection may affect salivary gland function, this has not been sufficiently substantiated or correlated with HAART, hence it has been herculean to distinguish salivary dysfunction as an integral process of the disease or a side effect of therapy. Also, compared with healthy counterparts, children living with HIV have lower salivary pH and flow rate [23, 24]. This may be partly attributed to early childhood medications that are mostly in syrup form, which may promote low-endogenous pH [25]. Even though, it has been reported previously that lowered salivary pH and flow rates are directly and significantly associated with increased caries incidence, [26, 27], how dental caries and these related salivary factors differ among children living with HIV and their exposed and uninfected children remain unclear as they have rarely been compared in these populations. Therefore, the present study seeks to investigate this gap.

The objective of this study is to examine the impact of perinatal HIV exposure (with or without infection) on salivary pH and flow rate and also, to determine the influence of these salivary properties on their dental caries experience. We hypothesize that there is a statistically significant difference in salivary functional properties to explain the differential dental caries experience in school-aged children living with HIV in Nigeria. It is unclear what impact perinatal exposure alone has on these properties, hence the inclusion of HEU children (given the growing population in sub-Saharan Africa).

## Materials and methods

### Study population

A total of 266 children were included in the study aged 4–11 years, comprising (1) HIV infected (HI) (*n* = 87); (2) HIV-perinatally exposed and-uninfected (HEU) (*n* = 82); and (3) HIV-unexposed and uninfected (HUU) (*n* = 97). The participants were part of a cohort study (DOMHaIN) and the recruitment has been previously described [18]. Parents of children between the ages of 4–11 years old attending the pediatrics PEPFAR outpatient clinic of the University of Benin Teaching Hospital (UBTH) HIV clinic were recruited for the HI and HEU groups, and the pediatrics out-patient clinic was leveraged for the HUU groups [18, 28]. Every guardian and parent who showed a willingness to be engaged in the study was taken through a written and verbal description of the study, in a one-on-one setting, and thereafter signed consent. A convenience subset of the DOMHaIN cohort with available salivary flow data and pH were included in this study. There were no significant differences in demographics of those included in the subset vs. those not included. The results of this study were based on a cross-sectional analysis of salivary properties and caries experience. This study was conducted over a period of one year.

### Ethical considerations

Before the commencement of the study, ethical approvals from the Institutional review boards at UBTH (ADM/E22/AVOL.VII/14,713, 31/1/2019), University of Maryland Baltimore (HP-00084081) and Rutgers State University of New Jersey (Pro2019002047), were obtained. All procedures performed in studies involving human participants were following the tenets of the Declaration of Helsinki. All the study activities, benefits/risks of voluntary participation, and withdrawal from the study were verbally communicated to parents/guardians or caregivers in English and or pidgin (broken) English. To confirm comprehension, questions were asked, and then written informed consent was obtained before recruitment.

All participants whose parents gave written informed consent to participate in the study after the study’s objectives were duly explained to them and were well understood by them. Children 4–11 years and above who gave consent to participate were recruited in the study.

Human subject protection measures were put in place for this study. Measures included using password-protected computers and systems for data collection, deidentified data and sample/specimen labels (where link/key only to study coordinators) and recruiting well-trained (HIPAA and human subject training) research staff/interviewers. The parents or legal guardians of participants signed a parental consent form and child assent was obtained.

### Examiners calibration

The calibration of the three examiners (dentists) ‘blinded to the participants’ groups was done with a pilot study conducted by a paediatric dentist in accordance with International Caries Detection and Assessment Scores (ICDAS). The three dentists examined ICDAS scores in a pilot study of ten participants, and independently by an experienced examiner. The results for each dentist were analyzed using Cohen’s Kappa statistics. The inter-examiner Kappa score was 0.91, and the intra-examiner values were 0.96, 0.92 and 0.84, respectively.

### Data collection and measures

Medical and dental history, demographic data, and oral health/caries assessment data were obtained with ‘a well-structured questionnaire from all participant’s parents or guardians in this study.

### Exposures of interest

The three exposure groups of interest examined in this study are the HI, HEU and HUU and they were categorized after undergoing the following screening. As previously described, we approached parents of children who were attending the pediatrics special treatment HIV clinic for the recruitment into this study and classified them as HI. Children were identified as HIV exposed but uninfected (HEU) if they were born to mothers previously diagnosed with HIV during or before pregnancy for that child. The HUU group were age- and sex-matched unexposed participants enrolled from pediatrics outpatient clinic. All participants were screened for HIV with serologic tests, where necessary, confirmatory PCR DNA tests of HIV were carried out. Our recruitment process was carried out by trained healthcare professionals who approached eligible participants’ mothers and provided them with both written and verbal descriptions of the study.

### Outcomes of interest

#### pH determination

The saliva pH of each participant was taken with the aid of MColourpHast™ (pH-indicator strips (non-bleeding) pH 0–14 universal indicator). Unstimulated saliva was collected from the floor of the mouth using a plastic pipette and transferred into a falcon tube and the four-colours shade embedded pH 0–14 deep strip was dipped into the sample and timed for 2 min with a digital timer. The colour change on the pH strip was matched at the end of the 2 min with the colour meter on the strip pack ranging from 0 to 14, and the pH value of the colour match was recorded accordingly.

#### Salivary flow rate determination

Participants were asked to lie on the dental chair after rinsing their mouths with water to remove food debris, a single wooden spatula was used as a bite prop to allow saliva to pool at the floor of the mouth for 5 min. After that, the whole unstimulated saliva was collected from the subjects using the suction method aided by a sterile plastic pasture pipette into a graduated 15 ml Falcon tube and the volume was measured and recorded. The salivary flow rate was documented as volume (ml) per time (minute).

### Confounding variables

We considered the following variables as the confounding factors; age, sex, gingival inflammation status, and CD4 + lymphocyte counts in our analysis. Gingival inflammatory status was scored using the Gingival index by Loe and Silness [29]. The colour, consistency and ease of bleeding on probing of their gingiva were assessed to arrive at a score.

### Caries assessment

Caries detection was based on modified ICDAS criteria and children were categorized as either non-affected (caries free) or affected based on the presence or absence of at least one carious lesion on any tooth surface in the mouth. dmft/DMFT indices were used to define quantitatively the caries severity. dmft/DMFT represents the sum of cavitated caries lesions, missing (due to caries), or restored (“filled”) tooth surfaces for primary/permanent dentition respectively. Distinct dmft, DMFT and their combined scores were recorded for each participant.

### Statistical analysis

Data was entered into and analyzed with SPSS version 21 and R version 4.1.2. The presentation was done with tables and graphs as appropriate. Frequencies, as well as mean and standard deviation, were used to describe numeric parameters. The distribution of gender across the groups was compared using ‘Fisher’s exact test, while ANOVA was used to compare pH and SFR among the groups. ‘Spearman’s correlation was done to test for the association between pH and SFR and other numeric variables. The Mann-Whitney U Test was carried out to compare pH and SFR between the gender groups. A *p*-value of less than 0.05 was considered statistically significant.

## Results

Overall, the mean (SD) age of enrolled participants was 7.4 (2) years and the distribution did not significantly differ across the groups (*p* = 0.13). Considering other demographic characteristics, groups did not differ by gender, delivery modes, gestational age and their current weight and height (Table 1).

**Table 1:** Description of the Population of Participants

	HI (N=87)	HEU (N=82)	HUU (N=97)	Overall (N=266)
**Gender**				
Female Male	43 (49.4%) 44 (50.6%)	35 (42.7%) 47 (57.3%)	45 (46.4%) 52 (53.6%)	123 (46.2%) 143 (53.8%)
**Age (in months)**				
Mean (SD) Median [Min, Max] [Q1, Q3] (IQR)	92.4 (24.5) 96.0 [41.0, 126] [79.0, 114] (34.5)	89.0 (22.2) 88.0 [42.0, 126] [74.0, 110] (35.8)	84.2 (24.1) 88.0 [42.0, 125] [62.0, 104] (42.0)	88.4 (23.8) 91.0 [41.0, 126] [69.3, 110] (40.5)
**Delivery Method**				
Caesarean Vaginal	9 (10.3%) 78 (89.7%)	16 (19.5%) 66 (80.5%)	23 (23.7%) 74 (76.3%)	48 (18.0%) 218 (82.0%)
**Premature Birth (gestational age < 36 weeks)**				
No	84 (96.6%)	77 (93.9%)	94 (96.9%)	255 (95.9%)
Yes	3 (3.4%)	5 (6.1%)	3 (3.1%)	11 (4.1%)
**Birth weight (in kg)**				
Mean (SD) Median [Min, Max] [Q1, Q3] (IQR) Missing	2.96 (0.584) 3.00 [0, 4.50] [2.50, 3.30] (0.800) 6 (6.9%)	2.96 (0.664) 3.00 [1.15, 4.90] [2.50, 3.40] (0.900) 0 (0%)	3.18 (0.537) 3.25 [1.50, 4.50] [2.90, 3.50] (0.600) 0 (0%)	3.04 (0.601) 3.00 [0, 4.90] [2.60, 3.50] (0.900) 6 (2.3%)
**Did Child Breastfeed?**				
No Yes	9 (10.3%) 78 (89.7%)	32 (39.0%) 50 (61.0%)	1 (1.0%) 96 (99.0%)	42 (15.8%) 224 (84.2%)
**Duration of Breastfeeding (in months)**				
Mean (SD) Median [Min, Max] [Q1, Q3] (IQR) Missing	12.4 (5.15) 14.0 [1.00, 24.0] [9.50, 15.0] (5.50) 8 (9.2%)	7.65 (4.83) 6.00 [1.00, 24.0] [6.00, 10.0] (4.00) 33 (40.2%)	14.4 (4.59) 15.0 [2.00, 28.0] [12.0, 16.0] (4.00) 0 (0%)	12.2 (5.46) 14.0 [1.00, 28.0] [6.00, 15.0] (9.00) 41 (15.4%)
**Was Mother on ART during pregnancy?**				
No Yes	77 (88.5%) 10 (11.5%)	13 (15.9%) 69 (84.1%)	NA NA	90 (53.3%) 79 (46.7%)
**Child’s Duration on ART (in months)**				
Mean (SD) Median [Min, Max] [Q1, Q3] (IQR) Missing	56.9 (46.7) 55.0 [0, 175] [5.00, 94.0] (89.0) 28 (32.2%)	97.2 (54.1) 103 [0, 200] [72.0, 138] (66.0) 1(1.2%)	NA NA NA 97 (100%)	80.2 (54.7) 84.0 [0, 200] [33.0, 120] (87.0) 126 (47.4%)
**CD4 count (in cells/mm**^**3**^)				
Mean (SD) Median [Min, Max] [Q1, Q3] (IQR)	729 (404) 640 [109, 2110] [477, 864] (387)	904 (381) 817 [373, 2000] [593, 1080] (490)	839 (312) 799 [34.0, 1830] [631, 957] (326)	823 (371) 771[34.0, 2110] [573, 1000] (428
**CD4 count > 500 cells/mm** ^**3**^				
No	21 (24.1%)	4 (4.9%)	7 (7.2%)	32 (12.0%)
Yes	66 (75.9%)	78 (95.1%)	90 (92.8%)	234 (88.0%)
**Current weight (in kg)**				
Mean (SD) Median [Min, Max] [Q1, Q3] (IQR)	22.1 (5.63) 22.0 [12.0, 39.0] [17.5, 26.3] (8.75)	24.3 (7.91) 23.3 [13.0, 67.5] [20.0, 27.4] (7.38)	24.1 (6.61) 23.0 [13.0, 44.0] [18.5, 29.0] (10.5)	23.5 (6.80) 22.5 [12.0, 67.5] [18.6, 27.9] (9.25)

Table 1 also shows that most (78%) children were birthed through vaginal delivery, the HI group had a greater proportion of birth though vaginal delivery when compared to the HEU group (66%). Only 11 children of the entire population were born prematurely and considering the groups, HEU had the highest proportion (6.1%) of premature born children. As expected, among the groups, the HUU has the highest average birthweight (3.18 kg). Similarly, more children in the HUU group (99.0%) were breastfed, compared to 60.1% and 89.7% respectively for HEU and HI children; they were also breastfed for the longest time (14.4 months) compared to the other groups.

Considering duration on anti-retroviral therapy (ART) at the time of this study, more mothers of children in the HEU group were on ART during pregnancy compared to mothers of children in the HI group (84% vs. 11%). Expectedly, children in the HEU group had been on postnatal ART prophylaxis/therapy for a longer time (97.2 months) compared to their HI counterparts (56.9 months). Interestingly, the HEU group had a higher mean CD4 + lymphocyte count than their HUU counterparts, HI group had the lowest mean CD4 + count. HEU group also had up to 95% of their participants with CD4 + counts > 500 cells/mm^3^, followed by the HUU group (92.8%) and the HI group (75.9%).

**Fig. 1:**
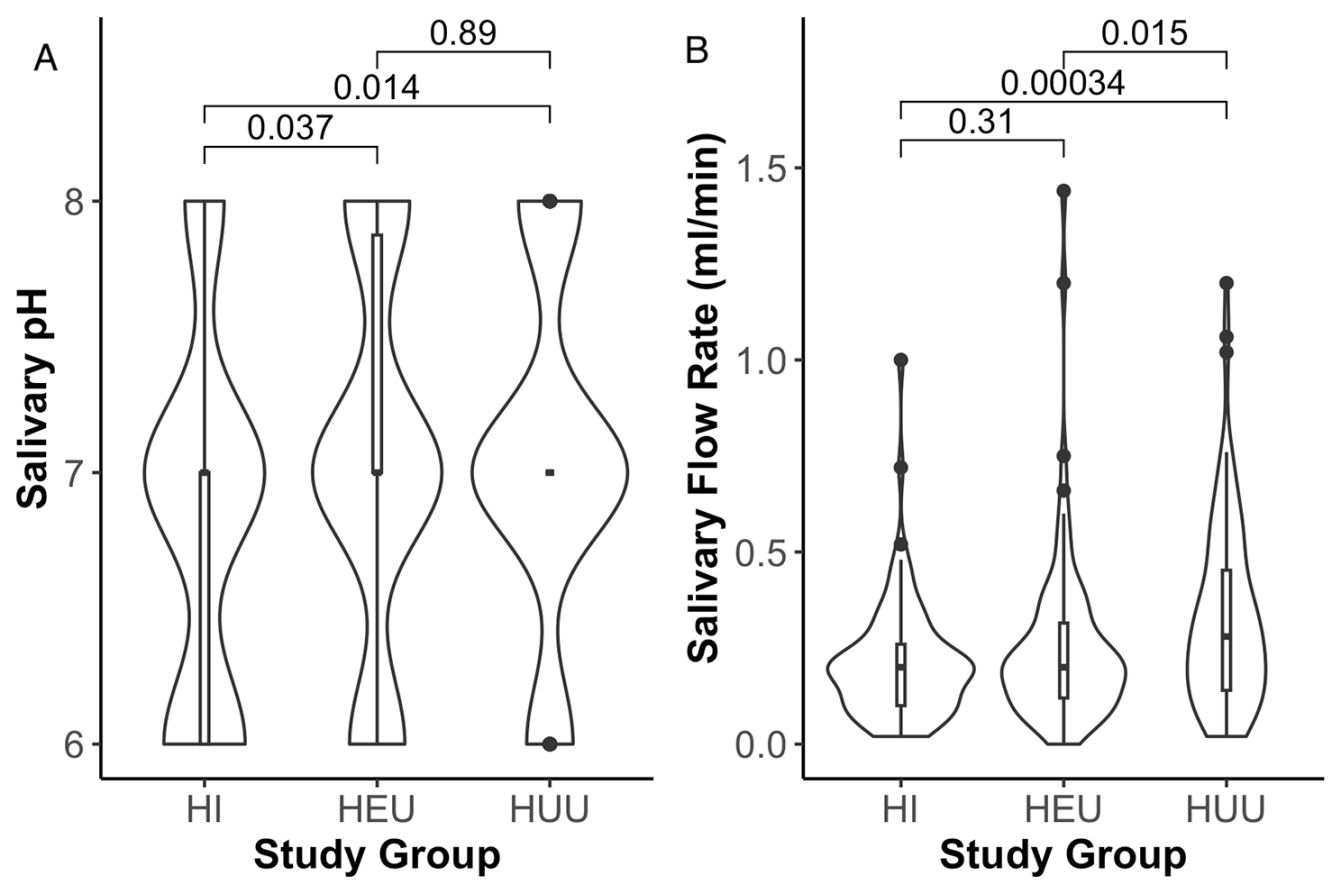
Salivary pH and Salivary Flow Rates in all groups. Both salivary pH **(A)** and salivary flow rate **(B)** are different across the three groups. There was a significant difference in salivary pH of HI vs. HEU (*p* = 0.037) and salivary flow rates of HEU vs. HUU (*p* = 0.015)

The difference in the distributions of pH among the study groups was statistically significant (*p* = 0.03). Across the groups, mean pH of the HI was significantly less than that of HEU and HUU (Fig. 1a). Similarly, as shown in Table 2, there was statistically significant difference in the SFR across the three groups (*p* = 0.004). For pairwise comparisons, the mean SFR of HUU differed significantly when compared to both the HI and HEU groups, however, there was no significant difference observed between HI and HEU groups (Fig. 1b).

**Table 2:** Distribution of Salivary pH and flow rates across the study groups

Parameter	HI (N = 87)	HEU (N = 82)	HUU (N = 97)	Overall (N = 266)	*P*- value
pH					
Mean (SD)	6.82(0.69)	7.04 (0.69)	7.06 (0.64)	6.98 (0.68)	0.033
Median [Min, Max]	7.00 [6.00, 8.00]	7.00 [6.00, 8.00]	7.00 [6.00, 8.00]	7.00 [6.00, 8.00]	
[Q1, Q3] (IQR)	[6.00, 7.00] (1.00)	[7.00, 7.88] (0.85)	[7.00, 7.00] (0)	[7.00, 7.00] (0)	
Salivary flow Rate					
Mean (SD)	0.21 (0.15)	0.26 (0.23)	0.33 (0.24)	0.27 (0.21)	0.004
Median [Min, Max]	0.20 [0.02, 1.00]	0.20 [0, 1.44]	0.28 [0.02, 1.20]	0.20 [0, 1.44]	
[Q1, Q3] (IQR)	[0.10, 0.26] (0.16)	[0.12, 0.32] (0.19)	[0.14, 0.45] (0.31)	[0.12, 0.36] (0.24)	
Missing	0 (0%)	0 (0%)	1 (1.0%)	1 (0.4%)	

Considering the risk factors affecting pH and SFR, the influence of perinatal HIV exposure and infection was evaluated in simple and multivariable linear regression models (Table 3). With respect to pH, HIV infection was a significant risk factor, when compared to HEU and HUU. On the other hand, HIV infection (HI) and exposure (HEU) were significant factors affecting their salivary flow rates when compared to their unexposed, uninfected (HUU) counterparts (Table 3). Other variables such as, gender, age and oral hygiene status expressed by the gingival inflammatory scores had no significant influence on the pH and SFR of study participants (Table 3). Worthy of note is that pH and SFR of males were higher than females (*p* = 0.07, *p* = 0.06 respectively).

**Table 3:** Summary of Risk Factors associated with Salivary pH and Flow Rate

	pH					
Participant Characteristics	Unadjusted			Adjusted^ψ^		
	Beta	(95%CI)	*p* value	Beta	(95%CI)	*p* value
**Study Group**						
HI	**-0.24**	**(-0.435, -0.045)**	**0.017**	**-0.23**	**(-0.172, -0.048)**	**0.028**
HEU	-0.02	(-0.217, 0.561)	0.850	-0.02	(-0.139, -0.015)	0.858
HUU	ref			Ref		
**Sex**						
Male	0.17	(0.0071, 0.332)	0.076	0.15	(-0.002, 0.102)	0.076
Female	ref			Ref		
**Age** (Continuous), in months	-0.0021	(-0.0055, 0.0013)	0.456	-0.0014	(-0.051, 0.052)	0.456
**log (CD4 lymphocyte count, /mm** ^**3**^ **)**	0.031	(-0.132, 0.194)	0.710	-0.002	(-0.024, 0.084)	0.983
**Gingival Index**	0.03	(-0.199, 0.263)	0.788	0.03	(-0.043, 1.04)	0.815
	**SFR**					
**Participant Characteristics**	**Unadjusted**			**Adjusted** ^**ψ**^		
	**Beta**	**(95%CI)**	***p value***	**Beta**	**(95%CI)**	***p value***
**Study Group**						
HI	**-0.11**	**(-0.172, -0.048)**	**0.000**	**-0.11**	**(-0.172, -0.048)**	**0.001**
HEU	**-0.07**	**(-0.131, -0.010)**	**0.025**	**-0.08**	**(-0.139, -0.015)**	**0.015**
HUU	ref			Ref		
**Sex**						
Male	0.05	(-0.463, 0.561)	0.062	0.05	(-0.002, 0.102)	0.061
Female	ref			Ref		
**Age** (Continuous), in months	0.0001	(-0.051, 0.052)	0.896	0.0005	(-0.051, 0.052)	0.418
**log (CD4 lymphocyte count, /mm** ^**3**^ **)**	0.03	(-0.025, 0.078)	0.313	0.03	(-0.024, 0.084)	0.272
**Gingival Index Score**	0.05	(-0.027, 0.118)	0.220	0.04	(-0.043, 1.04)	0.319

^Ψ^ multivariable linear regression analyses

**Fig. 2:**
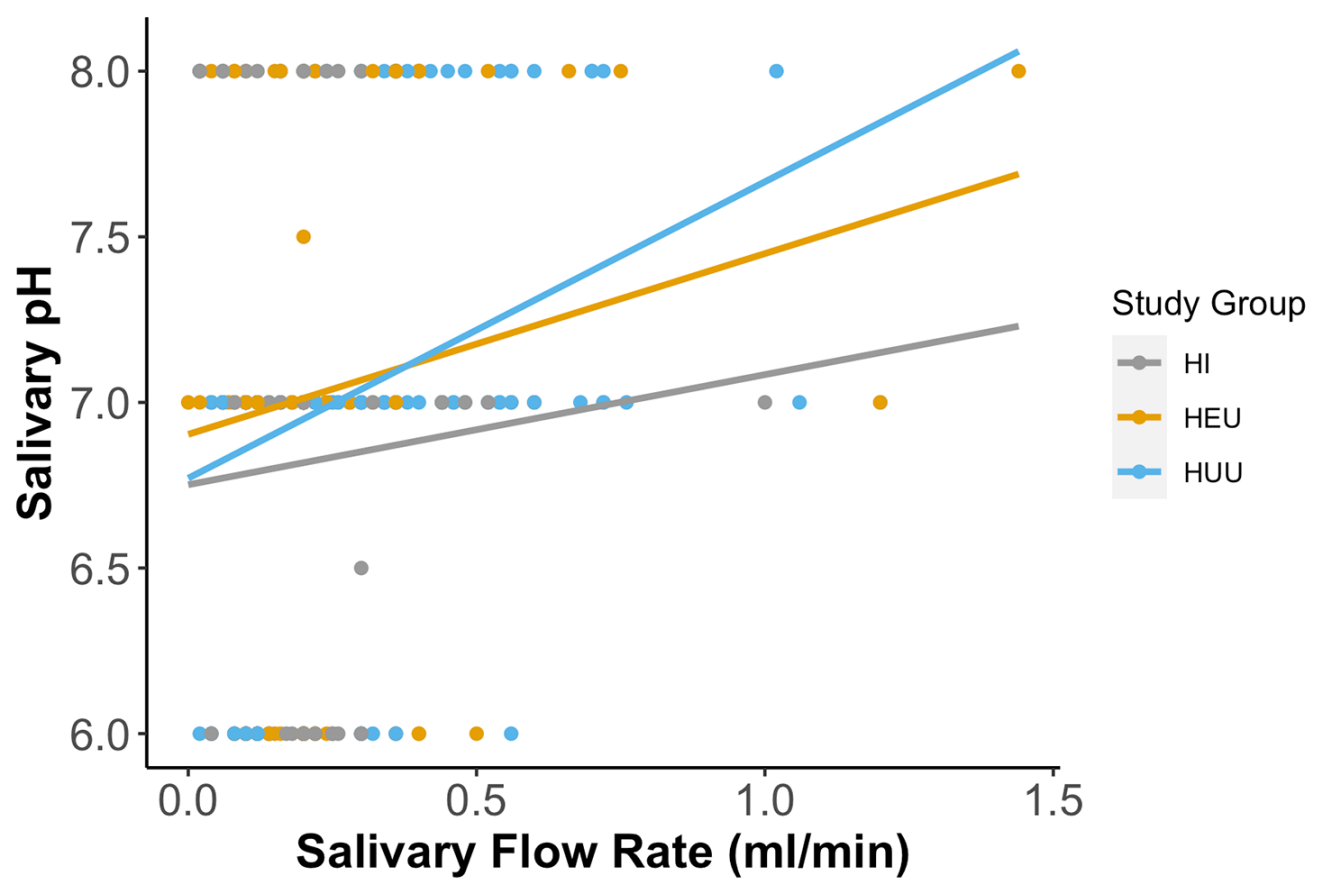
Relationship between Salivary pH and Flow Rates in all groups. Correlation coefficient for all groups (*r* = 0.229, *p* < 0.001); the HI group (*r* = 0.074, *p* = 0.498), HEU group (*r* = 0.178, *p* = 0.0111) and HUU group (*r* = 0.329, *p* = 0.001)

Considering the association of pH and SFR across the groups, overall, as well as in the HUU group only, there was a significant positive relationship between pH and salivary flow rate. This implied that an increase in pH was associated with an increase in salivary flow rate (Fig. 2). However, when observing the patterns in the three study groups, there was a significant relationship between the pH and salivary flow rate in all groups (*r* = 0.229, *p* < 0.001) as well as in HUU group (*r* = 0.329, *p* = 0.001). Conversely, there was no relationship between the pH and salivary flow rate in the HI group (*r* = 0.074, *p* = 0.498), and in the HEU group (*r* = 0.178, *p* = 0.0111).

**Fig. 3:**
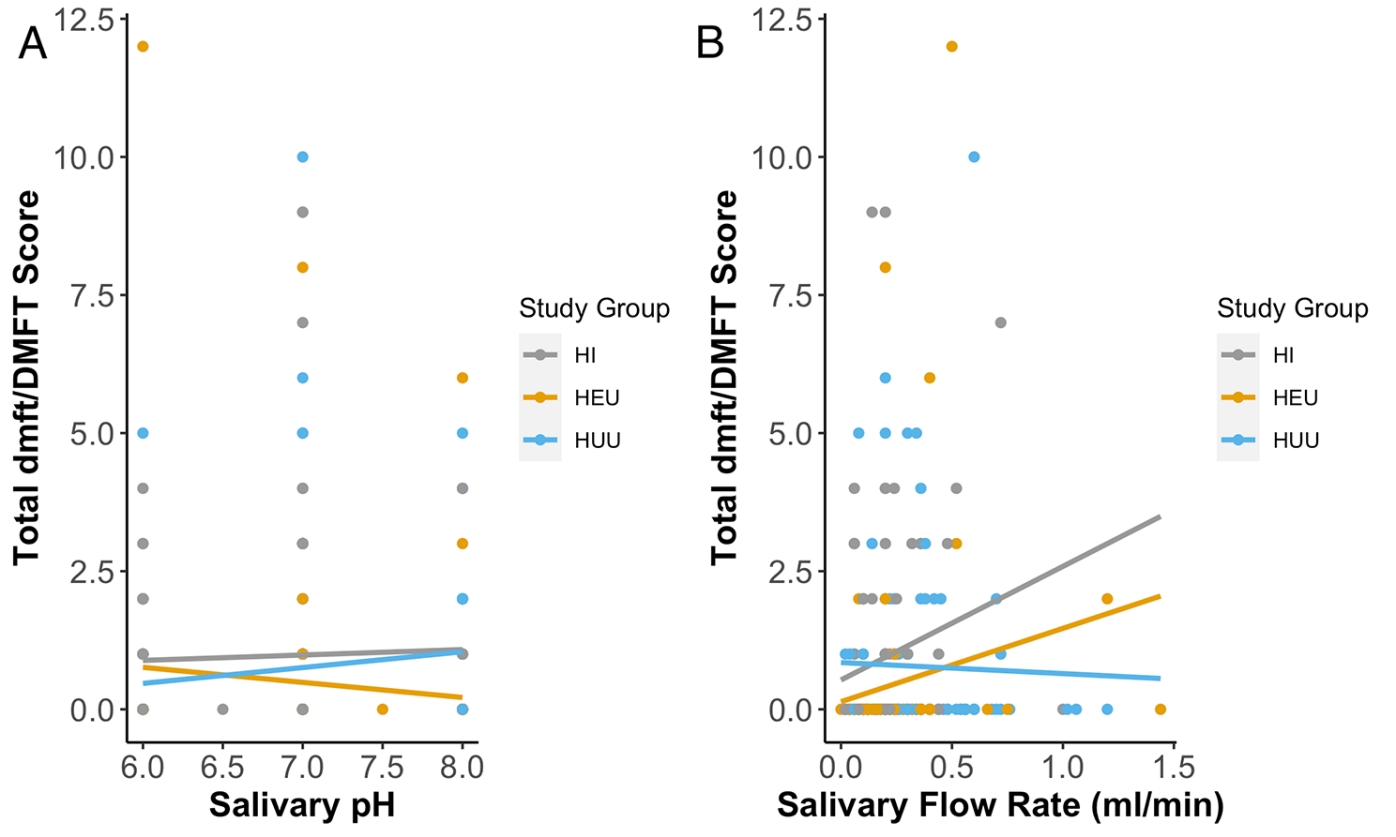
Scatter plots evaluating the relationship between dmft/DMFT and **(A)** salivary pH and **(B)** salivary flow rate for all study groups

The HI group had the highest mean DMFT of 0.87, while the mean within the HEU group was the lowest (Table 4). It is important to note that for HI and HEU groups, there was a rather unexpected positive correlation with increasing salivary flow rate; however, the relationship was weak and not significant (Fig. 3A and B). Across the groups, there was neither a significant relationship between DMFT and pH (Fig. 3A) nor between DMFT and SFR (Fig. 3B).

**Table 4:** Caries Prevalence and Severity Across Study Groups

Caries Attributes	HI (*N* = 87)	HEU (*N* = 82)	HUU (*N* = 97)	*p*-value*
Caries-affected in any teeth, n(%)	41(47)	20(21)	32(32)	0.006
Caries-affected in primary teeth, n(%)	31(36)	11(13)	27(28)	0.004
Caries-affected in permanent teeth, n(%)	18(21)	10(12)	5(5)	0.003
t, mean (sd)	2.5 (2.5)	3.1 (2.9)	2.9 (2.1)	0.69
DMFT, mean (sd)	2.5 (2.0)	1.9 (1.0)	1.2 (0.5)	0.25
Combined dmft/DMFT, mean (sd)	2.2 (1.8)	3.1(2.9)	3.0 (2.1)	0.28

*p value from ANOVA F statistic; sd – Standard Deviation; HI – HIV Infected; HEU - HIV Exposed and Uninfected; HUU - HIV Unexposed and Uninfected

## Discussion

This study examined the impact of perinatal HIV exposure and infection on salivary properties among school-aged children in Nigeria. To our knowledge, this study is among the first to compare all three groups (HI, HEU and HUU), particularly in the context of explaining the increased risk of caries in the HI group as previously reported by our group and others. Results from this study show that salivary properties with respect to the pH and SFR are impacted by perinatal HIV infection and exposure in school-aged children. In light of the rising population of children who are HIV-exposed but uninfected, this study leverages this unique group that has been shown to have suboptimal clinical outcomes compared to children born to uninfected mothers. We found that compared to HUU group, HEU group had similar pH levels but significantly less SFR.

Studies have shown that the properties of saliva, such as pH, flow rate and microbial composition, have been associated with dental caries to a large extent [30–32]. The pH of saliva is an essential component in maintaining the integrity of the oral cavity. After all, an increase in pH helps with the remineralization process, as it is well-documented that enamel dissolution occurs when the pH falls below the critical level [33, 34]. In this study, it was observed that there was a reduction in pH of participants in the HI group as compared to the HEU and HUU groups, which agrees with previous findings [23]. Although the exact mechanism behind the reduction of pH in the HI group is unknown, the impact of HIV on salivary pH has been well reported [35–37]. However, there are some postulations that early childhood medications including ART administered mostly in syrup form contain high amounts of sucrose and may foster low endogenous pH [25]. 

At rest, saliva flow helps keep the mouth moist and lubricates the mucous membrane [38]. Essentially, the greater the flow rate, the better the cleansing action of saliva on tooth surfaces and the lesser the chances of dental caries [34]. Although this study showed that the flow rates of HI did not differ significantly with that of HEU, the SFR in HI group was significantly less than that of HUU. This has also been seen in a previous study since salivary gland disease is a common manifestation among HIV-positive children receiving ART [23]. However, a US-based study, albeit with a larger and older children population reported higher, (though non-significant) salivary flow rates in HIV-infected children compared with uninfected and perinatally exposed groups [21, 23]. This may be attributed to the fact that these participants may have been on ART for a longer period. Also, another study that compared salivary flow rate between different durations of ART use reported a higher SFR in the group with the longest period of ART exposure [39]. 

This study and several other reports [11, 12, 21] that considered oral manifestations of HIV infection, incorporated the increasingly rising population of HEU children in this inquiry. The HEU group are unique in this cross-section because not only do they have poorer health outcomes generally, [14, 40] they also have an increased prevalence of developmental defect of enamel when compared to unexposed counterparts [28]. Additionally, children perinatally exposed to HIV and ART are likely to experience adverse peripartum consequences on tooth structure, and therefore need to be targeted for caries prevention strategies [12]. Interestingly, in agreement, the SFR of this unique group was significantly less than their uninfected counterparts.

There was a slight gender variation on pH and SFR in this study. Females had lower pH and SFR compared to males, although this difference did not reach significance. These findings are in line with previous reports that have attributed this gender variation to be associated with the colonization of bacterial community and secretory IgA, which are more in males than in females [41, 42]. The age of participants, their CD4 + counts at the time of recruitment and their gingival inflammatory scores did not significantly influence any of the salivary properties. This finding is in dissent to a previous report that attributes a significant decrease in SFR in periodontal diseases [43]. 

While the mean pH of each group was well above the critical enamel pH of 5.5, [44] HI group was closest (6.82 ± 0.67) to the critical pH for the dissolution of cementum and dentin [45, 46]. A previous study examining HI and HUU groups reported a comparable mean pH of 6.1 [23]. However, the reported DMFT of 4.0 contrasts substantially with the mean DMFT of HI (2.5), HEU (1.9) and HUU (1.2) groups. Perhaps this huge difference may be due to the differences in the demographics. Suffice to note that existing records infer an amplified risk of dental caries due to HIV infection or antiretroviral therapy [11, 20, 21, 47]. 

The significant differences in the duration of maternal gestational ART and postnatal ART exposure in HI and HEU groups may explain the subtle differences in the salivary properties of the HI and HEU groups, as in-utero factors also impact tooth susceptibility to caries. However, a recent finding by Lam et al., did not find any significant association between the caries experience and the use of ART in children living with HIV (CLWH) [9]. Considering this was a cross-sectional study, it couldn’t probe the long-term impact of maternal HIV infection on their functional salivary properties and caries experience. Nevertheless, the interim influence of perinatal HIV exposure and infection can be assessed within this design. Further, this study did not tackle the independent effect of other factors such as dietary history and oral hygiene practices, due to our inability to make inferences from questionnaires. Notwithstanding these limitations, the present study will constitute a basis for further quantitative and longitudinal studies on the influence of salivary factors on dental caries experience among children with perinatal exposure to HIV. We strongly recommend future related studies to evaluate the salivary biology of children exposed (uninfected) and infected with HIV.

## Conclusion

Perinatal HIV exposure and infection significantly impacts salivary pH and flow rate among school-aged children in Nigeria. Findings of this study implies that HIV infection influenced the salivary pH, while HIV maternal exposure (without infection) impacted salivary flow rates when compared to the unexposed. Furthermore, the salivary properties influenced their caries prevalence but not severity among these children.

## Data Availability

Medical and dental history, clinical data and associated dataset generated and/or analyzed for this current study cannot be made publicly available as required consent to publish data were not given. However, the corresponding author can make deidentified data available on reasonable request.

## References

[1] Fejerskov O (2004). Changing paradigms in concepts on dental caries: consequences for oral health care. Caries Res.

[2] Philip N, Suneja B, Walsh LJ (2018). Ecological approaches to dental caries prevention: paradigm shift or shibboleth?. Caries Res.

[3] Shitie A, Addis R, Tilahun A, Negash W. Prevalence of dental caries and its associated factors among primary school children in Ethiopia. Int J Dent. 2021;2021.

[4] Broadbent JM, Thomson WM (2005). For debate: problems with the DMF index pertinent to dental caries data analysis. Community Dent Oral Epidemiol.

[5] Moradi G, Mohamadi Bolbanabad A, Moinafshar A, Adabi H, Sharafi M, Zareie B (2019). Evaluation of oral health status based on the decayed, missing and filled teeth (DMFT) index. Iran J Public Health.

[6] Wang Y, Ramos-Gomez F, Kemoli AM (2023). Oral diseases and oral health-related quality of life among Kenyan children and adolescents with HIV. JDR Clin Trans Res.

[7] Ponnam SR, Srivastava G, Theruru K (2012). Oral manifestations of human immunodeficiency virus in children: an institutional study at highly active antiretroviral therapy centre in India. J Oral Maxillofac Pathol.

[8] Rajonson N, Meless D, Ba B (2017). High prevalence of dental caries among HIV-infected children in West Africa compared to uninfected siblings. J Public Health Dent.

[9] Lam PPY, Zhou N, Wong HM, Yiu CKY. Oral health status of children and adolescents living with hiv undergoing antiretroviral therapy: a systematic review and meta-analysis. Int J Environ Res Public Health. 2022;19(19). 10.3390/ijerph191912864 PMC9564723 36232165

[10] Kikuchi K, Furukawa Y, Tuot S, Pal K, Huot C, Yi S (2019). Association of oral health status with the CD4 + cell count in children living with HIV in Phnom Penh, Cambodia. Sci Rep.

[11] Akhigbe P, Chukwumah NM, Folayan MO (2022). Age-specific associations with dental caries in HIV-infected, exposed but uninfected and HIV-unexposed uninfected children in Nigeria. BMC Oral Health.

[12] Coker M, El-Kamary SS, Enwonwu C (2018). Perinatal HIV infection and exposure and their association with dental caries in Nigerian children. Pediatr Infect Dis J.

[13] Jumare J, Datong P, Osawe S (2019). Compromised growth among HIV-exposed uninfected compared with unexposed children in Nigeria. Pediatr Infect Dis J.

[14] Le Roux SM, Donald KA, Brittain K (2018). Neurodevelopment of breastfed HIV-exposed uninfected and HIV-unexposed children in South Africa. AIDS.

[15] Powis KM, Slogrove AL, Okorafor I (2019). Maternal perinatal HIV infection is associated with increased infectious morbidity in HIV-exposed uninfected infants. Pediatr Infect Dis J.

[16] Ramokolo V, Goga AE, Slogrove AL, Powis KM (2019). Unmasking the vulnerabilities of uninfected children exposed to HIV. BMJ.

[17] Filteau S (2009). The HIV-exposed, uninfected African child. Trop Med Int Health.

[18] Coker MO, Akhigbe P, Osagie E (2021). Dental caries and its association with the oral microbiomes and HIV in young children—Nigeria (DOMHaIN): a cohort study. BMC Oral Health.

[19] Shiboski CH, Yao TJ, Russell JS (2018). The association between oral disease and type of antiretroviral therapy among perinatally HIV-infected youth. AIDS.

[20] Birungi N, Fadnes LT, Engebretsen IMS, Lie SA, Tumwine JK, Åstrøm AN (2020). Caries experience and oral health-related quality of life in a cohort of Ugandan HIV-1 exposed uninfected children compared with a matched cohort of HIV unexposed uninfected children. BMC Public Health.

[21] Moscicki AB, Yao TJ, Ryder MI (2016). The burden of oral disease among perinatally HIV-Infected and HIV-Exposed uninfected youth. PLoS ONE.

[22] Mourad WF, Young R, Kabarriti R, Blakaj DM, Shourbaji RA, Glanzman J, Patel S (2013). 25-Year follow-up of HIV-positive patients with Benign Lymphoepithelial cysts of the parotid glands: a retrospective review. Anticancer Res.

[23] Kikuchi K, Yi S, Yasuoka J (2021). Oral health among HIV-positive and HIV-negative children in Phnom Penh, Cambodia: a cross-sectional study. BMJ Paediatr Open.

[24] Lauritano D, Moreo G, Oberti L (2020). Oral manifestations in HIV-positive children: a systematic review. Pathogens.

[25] Subramaniam P, Kumar K (2014). Cariogenic potential of medications used in the treatment of children with HIV infection. Spec Care Dentist.

[26] Cunha-Cruz J, Scott J, Rothen M, Mancl L, Lawhorn T, Brossel K (2013). Salivary characteristics and dental caries: evidence from general dental practices. J Am Dent Assoc.

[27] Rusu LC, Roi A, Roi CI, Victoria Tigmeanu C, Cosmina Ardelean L. The Influence of Salivary pH on the prevalence of dental caries. In: Laura-Cristina R and Lavinia CA, editors, Dental Caries -The Selection of Restoration Methods and Restorative Materials. IntechOpen; 2022. 10.5772/intechopen.106154.

[28] Onyia NE, Akhigbe P, Osagie E, Obuekwe O, Omoigberale A, Richards V (2023). Prevalence and associated factors of enamel developmental defects among Nigerian children with perinatal HIV exposure. J Clin Pediatr Dent.

[29] Löe H (1967). The Gingival index, the plaque index, and the retention index systems. J Periodontol.

[30] González-Aragón Pineda AE, García Pérez A, García-Godoy F (2020). Salivary parameters and oral health status amongst adolescents in Mexico. BMC Oral Health.

[31] Belstrøm D (2020). The salivary microbiota in health and disease. J Oral Microbiol.

[32] Stookey GK (2008). The effect of saliva on dental caries. J Am Dent Assoc.

[33] Inui T, Palmer RJ, Shah N, Li W, Cisar JO, Wu CD (2019). Effect of mechanically stimulated saliva on initial human dental biofilm formation. Sci Rep.

[34] Dodds M, Roland S, Edgar M, Thornhill M, Saliva (2015). A review of its role in maintaining oral health and preventing dental disease. BDJ Team.

[35] Ahmadi-Motamayel F, Amjad SV, Goodarzi MT, Poorolajal J (2017). Evaluation of salivary uric acid and pH in human immunodeficiency virus-infected patients: a historical cohort study. Infect Disord Drug Targets.

[36] Tjahajawati s, Sufiawati I, Rafisa A (2020). The correlation between salivary volume, salivary pH and CD4 in ARV and Non-ARV HIV patients. J Int Dent Med Res.

[37] Hegde MN, Malhotra A, Hegde ND (2013). Salivary pH and buffering capacity in early and late human immunodeficiency virus infection. Dent Res J (Isfahan).

[38] Tiwari M (2011). Science behind human saliva. J Nat Sci Biol Med.

[39] Kumar JV, Baghirath PV, Naishadham PP, Suneetha S, Suneetha L, Sreedevi P. Relationship of long-term highly active antiretroviral therapy on salivary flow rate and CD4 count among HIV-infected patients. J Oral Maxillofac Pathol. 2015 Jan-Apr;19(1):58–63. 10.4103/0973-029X.157203 PMC4451670 26097309

[40] Muhangi L, Lule SA, Mpairwe H (2013). Maternal HIV infection and other factors associated with the growth outcomes of HIV-uninfected infants in Entebbe, Uganda. Public Health Nutr.

[41] Ding T, Schloss P (2014). Dynamics and associations of microbial community types across the human body. Nature.

[42] Jafarzadeh A, Sadeghi M, Karam GA, Vazirinejad R (2010). Salivary IgA and IgE levels in healthy subjects: relation to age and gender. Braz Oral Res.

[43] Vallabhan CG, Sivarajan S, Shivkumar AD, Narayanan V, Vijayakumar S, Indhuja RS (2020). Assessment of salivary flow rate in patients with chronic periodontitis. J Pharm Bioallied Sci.

[44] Müller F, Zeitz C, Mantz H (2010). Elemental depth profiling of fluoridated hydroxyapatite: saving your dentition by the skin of your teeth?. Langmuir.

[45] Hoppenbrouwers PM, Driessens FC, Borggreven JM (1986). The vulnerability of unexposed human dental roots to demineralization. J Dent Res.

[46] Wefel JS (1994). Root caries histopathology and chemistry. Am J Dent.

[47] Kalanzi D, Mayanja-Kizza H, Nakanjako D, Sewankambo NK (2018). Extensive dental caries in a HIV positive adult patient on ART; case report and literature review. BMC Oral Health.

